# Optimizing Training Population Data and Validation of Genomic Selection for Economic Traits in Soft Winter Wheat

**DOI:** 10.1534/g3.116.032532

**Published:** 2016-07-20

**Authors:** Amber Hoffstetter, Antonio Cabrera, Mao Huang, Clay Sneller

**Affiliations:** Department of Horticulture and Crop Science, The Ohio State University Ohio Agriculture Research and Development Center, Wooster, Ohio 44691

**Keywords:** genomic selection, soft wheat, breeding values, GenPred, shared data resource

## Abstract

Genomic selection (GS) is a breeding tool that estimates breeding values (GEBVs) of individuals based solely on marker data by using a model built using phenotypic and marker data from a training population (TP). The effectiveness of GS increases as the correlation of GEBVs and phenotypes (accuracy) increases. Using phenotypic and genotypic data from a TP of 470 soft winter wheat lines, we assessed the accuracy of GS for grain yield, *Fusarium* Head Blight (FHB) resistance, softness equivalence (SE), and flour yield (FY). Four TP data sampling schemes were tested: (1) use all TP data, (2) use subsets of TP lines with low genotype-by-environment interaction, (3) use subsets of markers significantly associated with quantitative trait loci (QTL), and (4) a combination of 2 and 3. We also correlated the phenotypes of relatives of the TP to their GEBVs calculated from TP data. The GS accuracy within the TP using all TP data ranged from 0.35 (FHB) to 0.62 (FY). On average, the accuracy of GS from using subsets of data increased by 54% relative to using all TP data. Using subsets of markers selected for significant association with the target trait had the greatest impact on GS accuracy. Between-environment prediction accuracy was also increased by using data subsets. The accuracy of GS when predicting the phenotypes of TP relatives ranged from 0.00 to 0.85. These results suggest that GS could be useful for these traits and GS accuracy can be greatly improved by using subsets of TP data.

The improvement of high-throughput marker technology and the ability to generate inexpensive and abundant marker data can allow the use of molecular markers to reshape breeding and aid breeders in making faster genetic gains than using only phenotypic data. Traits of great economic importance in soft red winter wheat include grain yield, resistance to *Fusarium* Head Blight (FHB, causal agent *Fusarium graminearum*), and various quality traits. While QTL have been identified for these traits in soft red winter wheat (Cabrera *et al.* 2015; Hoffstetter *et al.* 2016; Liu *et al.* 2007, 2012; Smith *et al.* 2011) most have small effects suggesting that marker-assisted selection (MAS) for individual QTL will likely be ineffective.

An alternative to MAS is genomic selection (GS), which is a marker-based breeding tool that estimates all marker effects simultaneously and uses all markers to model the genomic estimated breeding value (GEBV) of individuals (Meuwissen *et al.* 2001). Genomic selection uses a training population (TP) that has been genotyped and phenotyped to develop a model that can be used to calculate the GEBVs of additional related individuals based solely on their genotype (Jannink *et al.* 2010). Individuals with desirable GEBVs can be used as parents to initiate a new cycle of breeding and to enter phenotyping trials. Genomic selection has been used in animal breeding to select sires and dams based on their GEBVs and is implemented routinely.

The use of GS in plant breeding has lagged behind its use in animal breeding in part because the commercial value in animal breeding is an individual’s breeding value whereas in crops the value is the individual’s performance *per se*. In addition GS requires genomewide, high-throughput, low cost genotyping of 1000s of individuals and this technology has been lacking for many crops. In wheat, studies have shown that GS can accurately model quantitative traits. In a soft red winter wheat population, Heffner *et al.* (2011b) found GS accuracy for grain yield was 0.20 while the accuracy of predicting flour quality traits and heading date was high (0.66 to 0.76). Huang *et al.* (2016) also studied GS accuracy in soft red winter wheat for agronomic and quality traits and reported GS accuracy ranging from 0.33 to 0.75. Other wheat studies have found prediction accuracy for grain yield ranging from 0.32 to 0.64 (Crossa *et al.* 2010; Heslot *et al.* 2012; Poland *et al.* 2012). Others are working to determine the use of GS at improving disease resistance in wheat. The GS accuracy for predicting adult stem rust in wheat was 0.61 (Rutkoski *et al.* 2014) while the accuracy for FHB incidence and severity in wheat was 0.56 and 0.64, respectively (Rutkoski *et al.* 2012).

Research is being conducted to determine the optimum number of markers and lines in the TP to estimate GEBVs. Bernardo and Yu (2007) used simulations and found increasing the number of markers increased accuracy when heritability was high, but not when trait heritability was low. Empirical studies have also shown that increasing the number of markers can increase prediction accuracy. In both biparental and multiparental wheat populations, the highest prediction accuracy was found using the largest marker set (Asoro *et al.* 2011; Heffner *et al.* 2011a, b) though Lorenz *et al.* (2012) reported that increasing the number of markers did not result in increased accuracy. Most empirical studies also show that increasing the TP size improves prediction accuracy (Heffner *et al.* 2011a,b; Lorenz *et al.* 2012).

These studies have investigated the effect on GS accuracy of modifying the TP by randomly deleting (or adding) lines and markers while little research has been done on optimizing GS accuracy by systematically selecting or eliminating TP lines or markers. Isidro *et al.* (2015) optimized a TP of 1127 soft winter wheat lines by using three methods to eliminate TP lines. Their results showed that by using a systematic approach a small population can be created that produces a GS accuracy that is similar to that obtained by using a large population.

There has been research in animals comparing GS accuracy using high- and low-density SNP chips in GS as the cost of the high-density chips can limit population size (Weigel *et al.*, 2009). Weigel *et al.* (2009) used a population of Holstein bulls and reduced the number of SNPs from 32,518 to subsets of 300 to 2000 SNPs based on the absolute value of the allele effect or even spacing along the chromosome. Using the markers subset based on allele effects provided a higher accuracy than evenly spacing markers; however both produced somewhat lower accuracy than using all markers. Vazquez *et al.* (2010) used a similar approach and got similar results. Moser *et al.* (2010) reported that small subsets of markers selected based on allele effects gave a similar accuracy to that obtained using all markers. In a population of chickens, Abdollahi-Arpanahi *et al.* (2014) partitioned 350,000 markers into five groups based on the absolute value of their allele effect. Across all three traits the group of SNPs with the greatest allele effects provided comparable or higher accuracies than using all 350,000 SNPs. Schulz-Streeck *et al.* (2013) worked with two maize populations and formed subsets of markers based on consistency of marker effects over environments. They reported that even subsets with 50 markers could provide similar accuracy to that obtained using the full set of 768 markers.

A few studies have been conducted to determine the prediction accuracy of GS models built using the TP data to predict the phenotype of lines from independent data sets. Lorenz *et al.* (2012) evaluated the accuracy of GS models built using data from one or two populations to predict the third population for FHB resistance and deoxynivalenol concentration in barley. When using one population to predict another, the prediction accuracies ranged from 0.47 to 0.77 for FHB and from 0.41 to 0.58 for deoxynivalenol concentration. The combination of two populations to predict the third population resulted in the highest prediction accuracies for both traits (Lorenz *et al.* 2012). Little research has been published using phenotypic data from one environment to predict phenotypes in another environment.

Genomic selection will have the greatest impact on plant breeding when the TP has been optimized to produce the greatest GS accuracy and when phenotypic data from one set of environments can predict performance in a different set of environments. Our objectives were to (1) determine the prediction accuracy of different GS models for four economically important traits of SRWW, (2) determine the ability of the GS model built on TP data to predict the phenotype of different populations of SRWW with varying relationships to the TP, (3) determine the effect of using subsets of TP lines and markers on GS accuracy, and (4) assess the accuracy of a GS model built on data from one environment to predict performance in a different environment.

## Materials and Methods

### Plant material

Three populations were used in this study. The first population is the TP and was previously described by Hoffstetter *et al.* (2016). The TP consisted of 470 F_4_-derived lines from The Ohio State University SRWW breeding program and is derived from 47 biparental crosses involving 23 parental lines that were selected for desirable GY, FHB resistance, quality, and pedigree diversity (Supplemental Material, Table S1). Seed was available for 22 of the 23 parental lines and these 22 lines comprise the parental population (PP) (Table S2). The third population consisted of 94 F_4_-derived lines from The Ohio State University SRWW breeding program and is called the validation population (VP) (Table S3). Of the 94 VP lines, 85 share some pedigree relationship to the TP: these 85 are termed the VP2 set. Seventeen VP lines were half sibs with some of the TP lines and these 17 are termed the VP3 set.

### Phenotypic data

Phenotypic data for the TP were previously described by Hoffstetter *et al.* (2016). The TP was evaluated in the 2009–2010 and 2010–2011 growing seasons. For GY, lines were grown in an augmented design with one or two replications at three locations: the Northwest Agricultural Research Station near Custar, Ohio (N 41° 16.8’ W 83° 50.4’), the North Central Agricultural Research Station near Fremont, Ohio (N 41° 21’ W 83° 7.2), and the Ohio Agriculture Research and Development Center near Wooster, Ohio (N 40**°** 46.2’ W 81° 55.8). Grain yield was obtained using a plot combine and seed weight per plot was adjusted to 13% moisture. The FHB index ratings were collected from an inoculated and misted nursery during June 2010 and 2011 in Wooster, Ohio from a randomized complete block design of single row plots with three replicates each year. Index was determined by visually estimating the percentage of symptomatic spikelets in a sample of spikes at about Feekes stage 11, which was about 21 d after heading (Feekes 10.3). A sample consisted of a handful of spikes (∼15 heads per handful) and three areas per plot were sampled. The SE and FY traits were analyzed using the methods of Smith *et al.* (2011) on grain samples taken from one replicate from the Custar and Fremont Ohio locations in 2010 and 2011. For GS for FHB, SE and FY we used genotype means over all environments.

Phenotypic evaluation of the PP was conducted during 2010–2011 and 2011–2012 growing seasons. The PP lines were included in part of a larger trial that was grown in an augmented design with either one or two replications at the same three locations as the TP (Custar, Freemont, and Wooster, Ohio). In 2010–2011 there was one replicate at each location and in 2011–2012 there were two replicates in Custar and one replicate in Wooster, Ohio. The augmented design consisted of five blocks per replication each with 38 unique lines and two checks (‘Malabar’ and ‘Pioneer 25R47’). Planting and harvest dates as well as nitrogen application are the same as described by Hoffstetter *et al.* (2016) for the TP. Heading date, height, FHB, SE, and FY data were collected as described for the TP. The VP lines were phenotyped during the 2011–2012 growing season in Custar and Wooster, Ohio using an augmented design with one replicate in each environment. The design consisted of three blocks each with 37 unique lines and three checks per block (Pioneer 25R47, ‘Bromfield’, and Malabar). Planting and harvest dates as well as fertilizer applications were the same as described by Hoffstetter *et al.* (2016). Height, HD, and FHB were also recorded as described for the TP. The SE and FY data were analyzed on grain samples from the trial in Wooster, Ohio.

Best linear unbiased predictions (BLUPs) of line effects were obtained as described by Hoffstetter *et al.* (2016). For grain yield, BLUPs were obtained over all environments (GYA) and just the Wooster (GYW) environments for all populations. BLUPs were also obtained over the combined data of Custar and Fremont (GYN) environments for the TP only. The BLUPs for FHB, FY, and SE were obtained over all environments for all populations. To normalize the TP FHB data, a square root transformation was conducted and BLUPs of these values were obtained for all lines. Subsets of TP lines with low genotype-by-environment (GEI) variance and high heritability, as described by Hoffstetter *et al.* (2016), were also used for each trait. Within each trait, the GEI variance for each line was plotted from lowest to highest and the “elbow” of the curve was visually estimated and then further identified by evaluating the difference between the GEI variance for consecutive lines in the data file: the elbow occurs where the slope of the two lines changes and that occurs where the difference between consecutive lines increases. Lines having a GEI variance greater than the elbow point were eliminated from the analysis. This created a subset of lines for GYA, GYW, and GYN containing 400 lines, a subset for FHB containing 440 lines, and a subset for quality traits containing 447 lines.

### Genotypic data

DNA for genotyping was extracted from a sample of lypholized leaves collected from two 2-wk-old seedlings per line using a 96-well format (DNAeasy 96 Plant Kit, Qiagen Group, Valencia, CA). All three populations were genotyped using genotyping-by-sequencing technology by Triticarte Pty Ltd Yarralumla, ACT, Australia (http://www.triticarte.com.au). The genotyping produced 4858 biallelic SNP markers and 28,311 dominant DaRT markers that were scored in the TP. For most of the GS work we only used the 4858 SNPs. Of the 4858 SNPs, 2442 SNP markers were scored in common between the PP, VP, and the TP. When using the TP data to develop GEBVs for the VP and PP lines, we only used the 2442 common markers. All markers used in the analysis had a call rate of at least 70%. Missing data in the TP were imputed based on the average marker score of lines from the same cross. Missing values were imputed as 1 if the average genotype score for other lines from that family was greater than 0 or as −1 if the average was <0. Missing data in the PP and VP populations were imputed as the common allele.

Unique subsets of the 4858 SNPs and the 28,311 DaRT markers from the TP were made for each trait using results from an association analysis (Hoffstetter *et al.* 2016) that was conducted using the subsets of TP lines with low GEI for each trait. Marker set 1 was the same for all traits and consisted of all the 33,169 DaRT and SNP markers scored in the TP. Marker subset 2 varied by trait and contained markers that were significantly associated with the trait at *P* < 0.05. For GY, marker subset 2 contained 2902 markers with a *P* < 0.05 for GYW or GYN: the correlation of marker effects for GYN and GYW was *r* = 0.64 (Hoffstetter *et al.* 2016). Marker subset 3 varies by trait and for grain yield it included markers from subset 2 but excluded markers with a high residual variance. The residual variance of markers ranged from −1.42 to 1.69. Huber weights were used to remove markers with a high residual variance using the ‘MASS’ package and rlm of R.3.0.1. The criteria for marker subset 3 for FHB and quality differ from grain yield because marker effects were estimated over environments for these traits and not individual environments as for grain yield (*e.g.*, GYN and GYW). Marker subset 4 varied by trait and included markers associated with each trait at a *P* < 0.01. For GY, this subset included 604 markers with a *P* < 0.01 for GYN or GYW. Marker subset 5 varied by trait and contained markers with a *P* < 0.005 for each trait. Finally, marker subset 6 was created for grain yield only and included 362 markers with *P* < 0.01, a low residual variance, and the average of the absolute value of allele effects for GYN and GYW greater than 30 kg hectare^-1^. The number of markers in each set for each trait is shown in Table 1.

**Table 1:** Accuracy of genomic selection for grain yield (all environments, GYA; Wooster Ohio, GYW; Northwest Ohio, GYN), *Fusarium* Head Blight resistance (FHB), softness equivalence (SE), and flour yield (FY) using either all TP data (470 lines and 33,169 markers) or subsets of lines (*n* < 470) chosen for low genotype-by-environment interactions, and subsets of marker data (M2–M6) chosen based on significance criteria (see *Materials and Methods*)

Trait	M1	M2	M3	M4	M5	M6
No. markers for GYA, GYW, GYN	33169	2902	2524	664	293	362
GYA, *n* = 470	0.45	0.77	0.76	0.73	0.74	0.73
GYA, *n* = 400	0.43	0.82	0.82	0.81	0.79	0.79
GYW, *n* = 470	0.57	0.79	0.79	0.77	0.77	0.75
GYW, *n* = 400	0.35	0.60	0.60	0.57	0.55	0.57
GYN, *n* = 470	0.41	0.36	0.36	0.27	0.21	0.28
GYN, *n* = 400	0.33	0.77	0.73	0.73	0.70	0.59
GYW to predict GYN, *n* = 470	−0.07	−0.10	−0.09	−0.08	−0.06	−0.08
GYW to predict GYN, *n* = 400	0.13	0.31	0.32	0.33	0.35	0.37
GYN to predict GYW, *n* = 470	−0.10	−0.16	−0.15	−0.15	−0.14	−0.15
GYN to predict GYW, *n* = 400	0.13	0.28	0.29	0.29	0.30	0.37
No. markers for FHB	33169	1556	1031	286	134	
FHB, *n* = 470	0.35	0.64	0.62	0.62	0.58	—
FHB, *n* = 440	0.37	0.81	0.79	0.78	0.72	—
2010 FHB to predict 2011 FHB, *n* = 470	0.13	0.17	0.17	0.17	0.21	—
2010 FHB to predict 2011 FHB, *n* = 440	0.13	0.17	0.18	0.16	0.20	—
2011 FHB to predict 2010 FHB, *n* = 470	0.15	0.32	0.31	0.32	0.36	—
2011 FHB to predict 2010 FHB, *n* = 440	0.16	0.38	0.37	0.38	0.42	—
No. markers for SE	33169	1672	1133	330	151	
SE, *n* = 470	0.51	0.87	0.85	0.83	0.80	—
SE, *n* = 447	0.51	0.89	0.87	0.85	0.82	—
2010 SE to predict 2011 SE, *n* = 470	0.33	0.57	0.53	0.59	0.59	—
2010 SE to predict 2011 SE, *n* = 447	0.32	0.58	0.53	0.59	0.50	—
2011 SE to predict 2010 SE, *n* = 470	0.24	0.32	0.30	0.33	0.37	—
2011 SE to predict 2010 SE, *n* = 447	0.24	0.32	0.31	0.33	0.26	—
No. markers for FY	33169	1632	968	316	166	
FY, *n* = 470	0.62	0.91	0.88	0.87	0.84	—
FY, *n* = 447	0.62	0.91	0.88	0.87	0.84	—
2010 FY to predict 2011 FY, *n* = 470	0.56	0.70	0.68	0.7	0.70	—
2010 FY to predict 2011 FY, *n* = 447	0.59	0.74	0.72	0.74	0.74	—
2011 FY to predict 2010 FY, *n* = 470	0.47	0.49	0.49	0.49	0.49	—
2011 FY to predict 2010 FY, *n* = 447	0.48	0.50	0.50	0.5	0.50	—

Accuracy is shown for the trait itself using cross-validation and when using data from one set of environments to predict the phenotype from the other set of environments. Also shown is the number of markers in each marker subset for each trait.

### Population relatedness

The degree of relationship between the TP and the VP was calculated using the 2422 SNP markers scored in both the TP and VP. Relationship was calculated as a simple matching coefficient which is equal to the probability that alleles randomly sampled from two individuals are identical by state. The simple matching coefficient was calculated using PROC IML in SAS 9.1. The relationship between the VP and the TP was visualized using a principal component analysis (PCA) conducted in R 3.0.2 using the function ‘eigen’. The scores of the first and second principal components were plotted to create a PCA graph to show how the VP lines grouped with the TP lines.

### Genomic selection

Three GS models were used in the TP with the 4858 SNP markers: Ridge-Regression BLUP (RRBLUP), Bayesian LASSO, and Random Forest. For each model ten-fold cross-validation was used to estimate GEBVs and the accuracy of GS. For cross -validation the TP lines in the prediction set were randomly selected without replacement allowing GEBVs of all 470 lines to be predicted within one cycle. Each model was implemented in R 3.0.2 (R Development Core Team 2011) using a Windows 2008 64-bit virtual machine with a 3.46 GHz Intel(R) Xenon(R) CPU processor and 32.0 GB of RAM.

RRBLUP was conducted using the ‘rrBLUP’ package 4.2 in R (Endelman 2011). The function mixed.solve was used, which uses a mixed model in the form of:

y=Xβ+Zu+ε

$$u∼N(0,K\sigma_{u}^{2}),$$

where **X** is the design matrix for the fixed effects β, **Z** is the design matrix for the random effects *u*, *u* are the random effects, **K** is a positive semidefinite matrix that accounts for the relatedness among individuals based on markers, and $\sigma_{u}^{2}$ is the additive variance (Endelman 2011). The residuals are normal with a constant variance and variance components were estimated by REML using the spectral decomposition algorithm of Kang *et al.* (2008). A marker-based formulation was used to estimate GEBVs for each marker, which were then multiplied by the marker matrix to give the resulting GEBVs of the individuals in the prediction set. Bayesian LASSO predictions were performed using the ‘BGLR’ package for R, version 1.0.4, and hyper-parameters chosen based on the guidelines of Perez *et al.* (2010). The residual variance was calculated using ${\text{df}}_{\varepsilon }$ = 3 and the equation:

$$S_{\varepsilon }=V_{\varepsilon }({\text{df}}_{\text{ε}}+2),$$

where $V_{\varepsilon }$ was chosen to reflect the expectation of the model’s residual variance. To calculate the variance of the infinitesimal effect of the ${\text{df}}_{\text{ε}}$ = 3 and the equation:

$$S_{u}=\frac{V_{u}({\text{df}}_{\varepsilon }+2)}{\bar{a}},$$

where $\bar{a}$ is the average of the diagonal value of **A**, the kinship matrix, and $V_{u}$ is the prior expectation of $\bar{a}\sigma_{u}^{2}$. Finally, lambda was calculated with the following equation:

$$\hat{\lambda }=\sqrt{2\sigma_{\varepsilon }^{2}V_{L}^{-1}\overset{p_{L}}{\underset{j}{\sum }}x_{Lj}^{\_2},}$$

where $\sigma_{\varepsilon }^{2}V_{L}=1$ and $\overset{p_{L}}{\underset{j}{\sum }}x_{Lj}^{\_2}$ is the sum of squares (over markers) of the average genotype (Perez *et al.* 2010). Random Forest predictions were made using the ‘randomForest’ package version 4.6-10 in R (Liaw and Wiener 2002). The RRBLUP and Bayesian LASSO models were conducted using 1500 cycles while the Random Forest model was conducted using only 500 cycles due to computational intensity. All six traits in the TP were analyzed (GYA, GYW, GYN, FHB index, SE, and FY) and FHB index was also transformed using a square root transformation to bring the data into normalcy (FHB_SQRT).

The accuracy of a GS model was determined using a Pearson’s correlation between the GEBVs of all lines and their phenotypic BLUP. The relative efficiency per cycle of GS as compared to a cycle of phenotypic selection (RE_c_) was calculated as rH where *r* is the accuracy of the model and *H* is the heritability of the trait (Hoffstetter *et al.* 2016). Relative efficiency of GS per year (RE_y_) was determined by multiplying the RE_c_ by the ratio of years to complete one cycle of phenotypic selection *vs.* years to complete one cycle of GS for each trait. In our program it is estimated to take 7 yr for one cycle of phenotypic selection for grain yield and 5 yr for one cycle for FHB, FY, and SE. One cycle of GS can be completed in 1 yr in winter wheat. Finally, proc.time was used to determine the length of time to complete 300 cycles of 10-fold cross-validation for each GS model.

The six marker subsets described above were used to create GS models for each trait using RRBLUP. We then used 10-fold CV to determine the accuracy for GS for each trait and marker subset. Marker subsets were used to build GS models using the TP data and then determine the accuracy of GS for each marker subset/trait combination using either all 470 TP lines or the subsets of lines with low GEI.

A model was built using the 2442 SNPs that were scored in common in the TP, PP, and VP and used to calculate the GEBVs of the VP and PP lines. These GEBVs were then correlated to the phenotypes of the PP and VP lines. Phenotypic information from the TP together with markers scored in common between the TP and PP and RRBLUP were used to calculate the GEBVs for the 21 PP lines. The TP is composed of progeny of the PP lines therefore the true breeding value (TBV) of each of the PP lines could be calculated to assess the ability of the GS model to predict the TBVs. The TBV of the ith PP line was calculated for each trait using the equation:

$$TBV_{i}=2(\mu^{\prime }-\mu )$$

where $\mu^{’}$ is the mean of the offspring of the ith parent, and μ is the mean of the TP. Both the phenotype and TBV of the PP lines were then correlated to their GEBV using unweighted and weighted correlations. A weighted correlation was used to account for the fact that some PP lines contribute more parentage to the TP than others. The weighted correlation used the proportion of TP parentage from each PP line as a weighting factor and was calculated using the equation:

$$r=\frac{\sum^{\text{​}}[w*\left(x-\bar{x}\right)*(y-\bar{y})]}{\sqrt{\left[\sum^{\text{​}}{\left(x-\bar{x}\right)}^{2}*w]*[\sum^{\text{​}}{\left(y-\bar{y}\right)}^{2}*w\right]}}$$

where *w* is the weight of each parent, *x* is the GEBV for each PP, $\bar{x}$ is the mean GEBV of all PPs, *y* is the phenotype or TBV of each PP, and $\bar{y}$ is the mean phenotype or TBV of all PPs.

The RRBLUP model was retrained using the TP phenotypic and data from markers that were scored in both the TP and the VP to obtain GEBVs of each VP line using the same method as described for the PP population. The prediction accuracy was determined using a Pearson’s correlation between the GEBVs of the VP lines and their phenotypic BLUPs. Accuracy was determined using the entire VP data set, as well as for the VP2 and VP3 subsets.

### Data availability

The authors state that all data necessary for confirming the conclusions presented in the article are represented fully within the article.

## Results

### Phenotypic data

Phenotypic data from the TP were previously described by Hoffstetter *et al.* (2016) and are briefly recapped here for convenience. For GY, each of the environments (location/year combination) produced a wide range of values for each trait. The GEI pattern among the six test sites showed that the two Wooster environments clustered together and provided different results than the Northwest Ohio (Custar and Fremont) environments (Hoffstetter *et al.* 2016). The entry-mean heritability for yield over all environments (GYA) was 0.60, in the Wooster environments (GYW) was 0.69, and in the Northwest environments (GYN) was 0.51. Heritability was 0.59, 0.85, and 0.92 for FHB, FY, and SE, respectively. For all traits there were lines in the TP, PP, and VP with numerically superior phenotypes to the elite check for each trait. Thus like the TP, the PP and VP can be considered elite, adapted soft red winter wheat populations.

### Training population (TP) cross-validation

Using only RRBLUP we obtained nearly identical accuracy using all 33,169 markers or just the 4858 SNPs. We decided to use just the 4858 SNPs to compare the accuracy of GS with different models as some models can take far too much time with 33,169. Using 10-fold cross-validation the RRBLUP and Random Forest models had the highest accuracies for all traits and produced nearly identical results (Table 2). The Bayesian LASSO model had the lowest accuracy for all traits with the exception of GYW. Excluding the Bayesian LASSO results, GS accuracy ranged from 0.34 to 0.63. Accuracies for GYA and GYN were significantly lower than for GYW. We determined which model was computationally faster for estimating GEBVs in the TP. When using all TP lines, 4858 markers, 300 iterations, and 10-fold CV, RRBLUP was fastest completing the process in 5.6 hr, which was 28.8 hr less than Random Forest and 80 hr less than Bayesian LASSO. Only results from using RRBLUP will be discussed further.

**Table 2:** Genomic selection accuracy (*r*), standard deviation of accuracy (σ), relative efficiency per cycle (RE_c_), and relative efficiency per year (RE_y_) of three genomic selection models using a TP of 470 wheat lines and 10-fold cross-validation for four traits: grain yield over all six environments (GYA), grain yield at Northwest Ohio environments (4 environments, GYN), grain yield at Wooster, Ohio (2 environments, GYW), flour yield (FY), softness equivalence (SE), and *Fusarium* Head Blight index (FHBI)

	Ridge-Regression BLUP*^a^*				Random Forest				Bayesian LASSO			
Trait	*r**^b^*	σ	RE_c_*^c^*	RE_yr_*^d^*	*r*	σ	RE_c_	RE_yr_	*r*	σ	RE_c_	RE_yr_
GYA	0.45	0.01	0.58	4.1	0.48	0.01	0.62	4.3	0.12	0.02	0.15	1.1
GYN	0.41	0.01	0.58	4.1	0.42	0.01	0.59	4.1	0.14	0.02	0.20	1.4
GYW	0.57	0.01	0.67	4.7	0.57	0.01	0.69	4.8	0.57	0.01	0.68	4.8
FY	0.62	0.01	0.67	3.4	0.63	0.01	0.68	3.4	0.22	0.01	0.24	1.2
SE	0.51	0.01	0.53	2.7	0.49	0.01	0.52	2.6	0.06	0.02	0.06	0.3
FHB	0.35	0.01	0.46	2.3	0.37	0.01	0.48	2.4	0.17	0.02	0.22	1.1

a RRBLUP and Bayesian LASSO (BLR) were run for 1500 cycles and RF was run for 500 cycles.

b Accuracy is the Pearson’s correlation between the phenotype and the genomic estimated breeding value.

c Relative efficiency per cycle calculated by r/√H.

d Relative efficiency per year calculated as RE_c_ times the ratio of years in a cycle of phenotypic selection to years in a cycle of genomic selection.

The relative efficiency of GS *vs.* phenotypic selection per cycle (RE_c_) and per year (RE_y_) was calculated. All assessments of relative efficiency showed GS to be more efficient than phenotypic selection. The greatest RE_y_ for any grain yield trait was found for GYW (4.8), which was the grain yield trait with the highest accuracy and the highest heritability.

### Using TP data to predict the value of other populations

We assessed the ability of GS to predict the phenotypes of related lines that were phenotyped in independent environments. We used RRBLUP and all TP data to calculate the GEBVs of the PP and VP lines and estimated accuracy by correlating those GEBVs with the phenotypes of the PP and VP lines using both unweighted and weighted correlations. The RRBLUP model was chosen because it was more efficient (lower computational time and equal or higher accuracies) than Random Forest or Bayesian LASSO. The accuracy of GS models based on the TP data to predict the PP phenotypes was considerably greater for all traits when we used a weighted correlation than an unweighted correlation (Table 3). The accuracy of GS ranged from −0.23 to 0.85 for weighted correlations. Accuracy was low for GYA and GYN but was 0.67 for GYW using the weighted correlation. On average, the GS accuracy for predicting the TBV of the PP was similar or lower than the accuracy for the PP phenotypes (Table 3). As with the phenotypes, accuracy was generally higher when using the weighted correlation than the unweighted correlation. Interestingly, the accuracy for GYA and GYN increased greatly when using TBV *vs.* phenotypes while the accuracy for SE and FHB was greatly reduced. The difference between the mean phenotype of the top three (T3) and bottom three (B3) PP lines showed that the T3 group was always superior to the mean of the B3 group for all traits and this difference was significant at *P* < 0.1 for GYW, SE, and FHB.

**Table 3:** Genomic selection accuracy obtained using data from the TP to calculate genomic estimated breeding values (GEBVs) for lines in the Parental (PP) and Validation (VP) populations and correlating those GEBVs to the phenotypes of the PP and VP lines

	PP – Phenotypes		PP – True Breeding Values		VP – Phenotypes	
Trait	Unweighted*^a^*	Weighted	Unweighted	Weighted	All VP Lines	85 Most Related VP Lines
GYA	0.02	0.08	0.44	0.34	−0.17	−0.19
GYN	−0.41	−0.23	−0.02	0.16	−0.17	−0.16
GYW	0.57	0.67	0.32	0.31	−0.25	−0.27
FY	−0.15	0.50	0.13	0.55	0.05	0.05
SE	0.10	0.85	0.00	−0.22	0.27	0.33
FHB	0.14	0.47	−0.05	−0.03	0.22	0.22

For the PP we also correlated the GEBVs to the estimates of the TBVs of the PP lines. We used unweighted and weighted correlations in the PP: the weights were the percentage TP parentage that was derived from each PP line. In the VP the correlation was performed using all VP lines or just the 85 VP lines that had a pedigree relationship to the TP. The traits are grain yield over all five environments (GYA), grain yield at Northwest Ohio environments (GYN), grain yield at Wooster Ohio environments (GYW), flour yield (FY), softness equivalence (SE), and *Fusarium* Head Blight index (FHB).

a Accuracy is the Pearson’s correlation between the phenotype and the GEBV of the 21 parental lines.

In general all accuracies from using TP data to predict the GEBVs of the VP lines were low and all were lower than those observed when using the PP phenotypes and weighted correlations (Table 3). Assessing accuracy using data from just 85 VP lines that were related to the TP and PP did not impact GS accuracy. In the VP, only SE and FHB were moderately predicted by the TP data with accuracies ranging from 0.22 to 0.33. There was no significant difference between the mean phenotype of the top three (T3) and bottom three (B3) VP lines as ranked by their GEBVs for any trait either (Table 4). The T3 group was superior to the B3 group for GYN, FY, SE, and FHB and this difference was significant for FY and FHB at *P* < 0.1.

**Table 4:** Average phenotype of the top three (T3) and bottom three (B3) of the 21 parental lines (PP) and validation population lines (VP) as ranked based on their genomic estimated breeding values (GEBVs) that were predicted using data from the training population (TP) and the Ridge-Regression BLUP model

	PP				VP			
Trait	Avg. T3	Avg. B3	T3-B3	P-value	Avg. T3	Avg. B3	T3-B3	P-Value
GYA	4782	4600	181	0.45	5100	5442	−343	0.52
GYN	4529	4450	78	0.67	4074	3955	119	0.34
GYW	5496	4616	880	0.06	5846	5908	−63	0.84
FY	69.0	68.9	0.13	0.92	69.0	64.4	4.6	0.02
SE	58.7	57.4	1.3	0.09	58.7	56.4	2.3	0.46
FHB	16.4	24.2	−7.8	0.07	9.6	16.5	−6.9	0.06

Values are shown for grain yield over all environments (GYA, kg hectare^-1^), grain yield at Northwest, Ohio environments (GYN), grain yield at Wooster, Ohio environments (GYW), flour yield (FY), softness equivalence (SE), and *Fusarium* Head Blight index (FHB).

**Figure 1:**
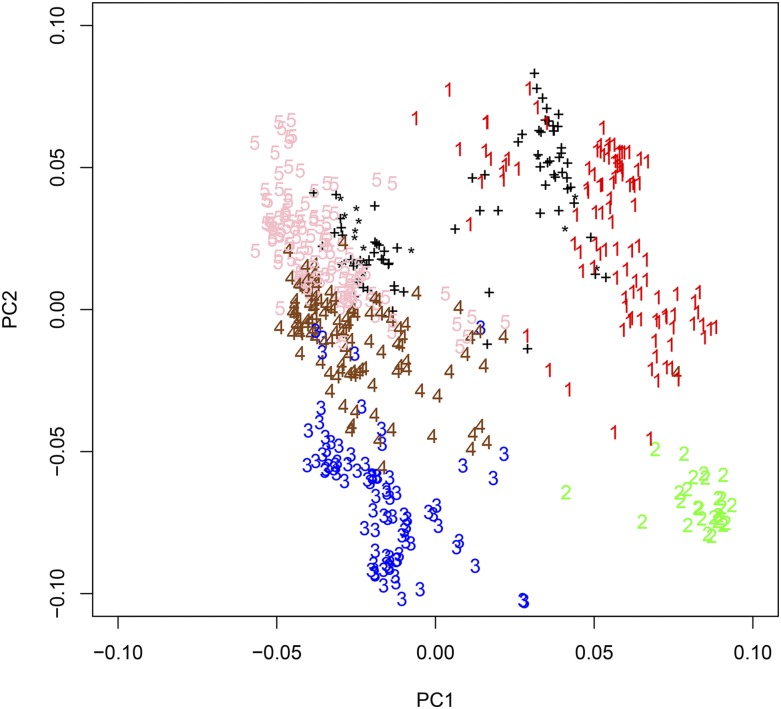
Principal component analysis of the 470 wheat lines of the training population (TP) and the 94 lines of the validation population (VP) using data from markers scored in both populations. Five groups of TP lines were defined by Cabrera *et al.* 2014 and are identified as groups 1 to 5. The VP are identified by the “+” and the “*” where the “+” represents the 85 lines with varying relationship to the TP and the “*” represent the 17 VP lines that are half sibs of the TP.

The results of the simple matching coefficient indicate the VP and the TP populations are related. The average genetic similarity within each population alone was 0.69, while the average genetic similarity between the VP and TP was 0.68. The PCA also shows a relationship between the VP lines and the TP lines (Figure 1).

### Accuracy of GS using subsets of TP data and cross-validation

The accuracy of the GS model was determined using data subsets derived from the TP by (1) removing TP lines with high GEI from the TP for each trait and (2) selecting markers based on results from an association analysis conducted using the TP data (Hoffstetter *et al.* 2016). In this analysis we used all 33,169 markers as they were used by Hoffstetter *et al.* (2016) in an association analysis in this population. Relative to using all TP data, the accuracy of GS for grain yield decreased 23% when using data from all markers and a subset of 400 lines with low GEI, though using only lines with low GEI had little effect on accuracy for the other traits (Table 1).

The GS accuracy was increased when using data from all TP lines and subsets of significant markers for all traits except GYN (Table 1). Excluding GYN, GS accuracy for the marker data subsets increased relative to using all markers by 41% (FY) to 76% (FHB) with the largest gains for all traits occurring for marker subset 2. The other smaller marker subsets gave similar accuracies as marker subset 2. Marker subset 5 had the fewest markers for all traits with, on average, <1% of the markers in the full set, yet provided very similar accuracies to those of marker subset 2, and provided superior accuracy compared to the full set of markers for all traits (Table 1). For example, marker subset 5 for FHB had just 134 markers and had an accuracy (0.58) that was similar to that of marker subset 2 (0.64) with 1672 markers and superior to the accuracy using the full set of 33,169 markers (0.35). We also evaluated GS accuracy using all nonsignificant markers (*e.g.*, all markers minus the subset 2 markers) for each trait. These subsets of nonsignificant markers produced within-environment GS accuracies that were on average 20% lower than the accuracy obtained using all marker data, and 46% less accurate than using marker subset 2 (Table S4).

There was a positive interaction between using subsets of TP lines and subsets of markers for GYA, GYN, and FHB where the greatest accuracies were obtained using the combination of a subset of lines with low GEI and marker subset 2: these accuracies were 75% (GYA), 113% (GYN), and 121% (FHB) greater than the accuracy obtained using all TP data (Table 1). For GYW, using just the subset of low GEI lines decreased accuracy, though using the subset of lines and the reduced marker sets resulted in accuracies that were similar to those obtained using all data (Table 1). The combination of subsets of low GEI lines and marker subsets did not increase GS accuracy for SE and FY above that obtained using just the reduced marker subsets.

### Using subsets of TP data to predict phenotypes between environments

Phenotypic data for grain yield in the TP from only one set of environments (*e.g.*, GYW data or GYN data) were used to build a GS model that was then used to predict the phenotype of the TP lines in the other set of environments. The accuracies of these between-environment predictions were low and negative when using all TP data (Table 1). Positive GS accuracies were obtained using the data from the subset of 400 lines with low GEI and all markers (Table 1), and was even further increased by using marker subsets (Table 1). The highest between-environment accuracy (*r* = 0.37) was found using the 400 low GEI lines and marker subset 6. This set contained 362 markers that had low residual variance, were significant for GYW or GYN, and the absolute value of allele effects for GYW or GYN were greater than 30 kg hectare^-1^. Similar, positive accuracies for between-environment predictions for grain yield were also found with the other marker subsets. The results were similar when using either GYW or GYN to train the model and predicting the other environment.

The TP phenotypic data for FHB, FY, or SE collected from 1 yr (2010 or 2011) were used to build a GS model that was used to predict the phenotype of the TP lines in the other year. When using all marker data the GS accuracy for between-year was low for FHB (range 0.13 to 0.16) and moderate for SE and FY (range = 0.24 to 0.59) when using either all TP lines or the subsets of lines with low GEI (Table 1). The GS accuracies remained low for FHB when using the marker subsets and 2010 data to build the model. The GS accuracies for FHB increased though when using the reduced marker subsets and 2011 phenotypic data to build the model: the accuracy using marker subset 5 and lines with low GEI, 0.42, was 180% greater than using all TP data (0.15).

For SE and FY, removing lines with high GEI did not increase prediction accuracy between years regardless of marker subset (Table 1). Using reduced marker subsets had little impact on between-year accuracy when using 2011 data to train the model to predict 2010 FY phenotypes, and produced a modest improvement for predicting 2010 SE phenotypes. Using the reduced marker subsets with 2010 TP data increased the ability to predict the 2011 phenotypes as GS accuracy increased by an average of 70% for SE and 26% for FY when compared to using all TP data.

We also evaluated GS accuracy using all nonsignificant markers (*e.g.*, all markers minus the subset 2 markers) for each trait. These subsets of nonsignificant markers produced between-environment GS accuracies that were on average 15% lower than the accuracy obtained using all marker data, and 33% less accurate than using marker subset 2 (Table S4).

## Discussion

### Cross-validation of GS using all TP data

The TP, PP, and VP all consisted of elite inbred soft red winter wheat breeding lines with adaptation to Ohio. The populations showed a range of phenotypes, with some lines in each population outperforming the best checks for each trait. The TP data were previously used to detect QTL for GY, FHB resistance, SE, and FY (Hoffstetter *et al.* 2016) though none of the QTL identified for these traits had a large effect suggesting the population would be better suited for GS for these traits.

For all traits except GYW, the RRBLUP and Random Forest GS models produced similar GS prediction accuracy while the Bayesian LASSO model produced the lowest prediction accuracy (Table 2). This is consistent with results from other studies which have found that shrinking all marker effects equally toward 0 and assuming they have a common variance produces similar or superior results to those models that do not impose this restriction such as Bayes A and Bayes B (Heffner *et al.* 2011a,b; Heslot *et al.* 2012; Lorenz *et al.* 2012). The results of this study also found the RRBLUP model to be computationally much faster than the other two models, therefore it was chosen as the model for all further data analysis. Further discussion will be restricted to the RRBLUP results.

The cross-validation accuracies from the GS models built using all TP data indicate that GS could be a useful tool within this population for these economically important traits. For grain yield the highest accuracy was found using data from the Wooster, Ohio (GYW) environment alone (Table 2). During the 2010–2011 growing season, some plots at the northern Ohio locations suffered from water damage (Hoffstetter *et al.* 2016). This resulted in a lower entry-mean heritability for GYN (*H* = 0.51) compared to GYW (*H* = 0.69) and likely explains why accuracy for GYN was lower than GYW. The accuracies for grain yield using all TP lines and marker data ranged from 0.41 to 0.57 (Table 2) and are generally similar to, and often higher than, the accuracy for grain yield reported by others. In a soft winter wheat multifamily population, Heffner *et al.* (2011b) reported accuracy for grain yield of 0.20 using RRBLUP as the prediction model. The prediction accuracy of RRBLUP for grain yield was 0.36 in another soft winter wheat population (Heslot *et al.* 2012). Combs and Bernardo (2013) reported a prediction accuracy of 0.10 for grain yield using the RRBLUP model in a wheat population. Prediction accuracies of 0.30 to 0.32 for grain yield were reported in a population of wheat lines from Chile, CIMMYT, and Uruguay using RRBLUP (Lado *et al.* 2013). Using two populations of wheat, Crossa *et al.* (2014) found the accuracy of GS for grain yield using RRBLUP ranged from 0.29 to 0.67 in a population of 306 lines, while in a population of 599 lines the accuracy was 0.42 to 0.45.

The GS accuracy for FHB using all TP data was 0.35, which is lower than most other reports for FHB resistance. In barley, the accuracy of GS for predicting the percentage of FHB infected kernels ranged from 0.41 to 0.67 (Lorenz *et al.* 2012) and for predicting FHB severity the accuracy was 0.30 to 0.39 using RRBLUP (Sallam *et al.* 2015). In soft winter wheat, Rutkoski *et al.* (2012) reported a prediction accuracy of 0.56 and 0.64 for FHB incidence and severity, respectively. The low accuracy of this study could be because there was low disease pressure in 2011 (Hoffstetter *et al.* 2016) even though the heritability for FHB in this study was moderate (H = 0.69). The accuracies of FY and SE with RRBLUP were 0.62 and 0.51, respectively (Table 2). Heffner *et al.* (2011b) reported a GS accuracy of 0.76 and 0.66 for FY and SE, respectively, in a multifamily soft wheat population using RRBLUP. The accuracy for FY and SE in a biparental population was 0.57 and 0.37, respectively (Heffner *et al.* 2011a). The accuracies reported for FY and SE in our study are in these ranges.

The relative efficiency per cycle (RE_c_) of GS across traits mostly reflected the GS accuracy. The RE_c_ for SE and FY in this study (0.53 and 0.68, Table 2) are comparable to those reported by Heffner *et al.* (2011a) in two biparental populations (0.29 to 0.70). Over all traits the RE_c_ values show that a cycle of GS is not as efficient as a cycle of phenotypic selection. However, the real advantage of GS is it can shorten the duration of a breeding cycle and improve gain per year (Goddard and Hayes 2007; Heffner *et al.* 2009, 2011b; Jannink *et al.* 2010). In this population the relative efficiency of GS on per year basis (RE_y_) ranged from 2.4 (FHB) to 4.8 (GYW, Table 2) which indicates that GS can be an efficient method for making genetic gains for these four traits by decreasing the breeding cycle time to 1 yr.

### Validating the TP GS model in related populations

The ability of the GS models built using all of the TP data to predict the phenotypes of other related lines varied by trait and population. The best results were obtained when predicting the phenotype of the PP lines as these lines were more related to the TP than were the VP lines. For most traits in the PP population, higher prediction accuracy was found using a weighted correlation than an unweighted correlation (Table 3). The reason the weighted correlation provides a higher accuracy then the unweighted correlations can be attributed to the fact that each PP line did not contribute equally to the parentage of the TP and weighting the correlation accounts for this. The models were less predictive of the TBV of the PP lines than it was of the phenotype of the PP lines. Because the TBV is estimated based on the performance of the progeny, the TBV of PP lines with few offspring in the TP is probably poorly estimated. In addition, the TBV of an individual should be estimated by mating the individual to a large sample of random individuals from the population. This was not the mating scheme used in this study, therefore it is likely that all estimated TBVs in this study have considerable bias. Overall the GS accuracies found for the PP range from −0.15 to 0.85 across all traits and analyses and shows that the performance of the PP lines can be estimated using GS models built using progeny (TP) data. To date, there is little research on using the progeny to predict the performance of parental lines. Sallam *et al.* (2015) used the parental lines to predict the performance of the progeny in a barley population. Using RRBLUP the accuracy for grain yield was 0.57. The accuracies found for GYW (0.57 and 0.67, Table 3) in this study are very close to those reported by Sallam *et al.* (2015).

The GS model built using the TP data were less successful at predicting the performance of the VP lines than the PP lines even though the VP and TP appeared quite related based on marker similarity. This marker-based similarity between the VP and TP occurred despite having only three immediate parents in common. The poor predictive performance for the VP sets may also be due to limited phenotyping of the VP as all phenotypes, except for FHB, were obtained from unreplicated trails. Also the VP lines were evaluated in a different year from the TP and therefore GEI may be affecting the ability to predict the performance of lines tested in another environment. Collectively, the results from the PP and VP show a GS model built using the TP data can predict the phenotypes of a highly related population, even when the phenotypes were obtained from different environments: the degree of this accuracy though varied by trait.

### Accuracy of GS when using subsets of TP data

Most studies and simulations show that increasing the size of the TP and the number of markers increases the accuracy of GS (Bernardo 2009; Bernardo and Yu 2007; Heffner *et al.* 2011b; Lorenz *et al.* 2012; Poland *et al.* 2012). In contrast, we show that the accuracy of GS can be greatly improved by systematically reducing the number of lines and markers in the TP data set. Compared to using all lines, removing TP lines with high GEI from the TP either reduced, or did not impact, prediction accuracy when we used all markers. When using all TP lines and systematically reducing the number of markers in the model to include only those markers significantly associated with the trait at *P* < 0.05 (marker subset 2), the prediction accuracy was increased for all traits except GYN (Table 1). Depending on the trait, marker subset 2 contained just 4.7–8.7% of all the markers. The gain in GS accuracy from using marker subset 2 with all 470 lines ranged from 39% to 83% (excluding GYN) compared to using all markers. In addition marker subset 5 had <1% of the TP markers for any trait and produced GS accuracies that were similar to those of marker subset 2 and that were superior to using all markers for all traits except GYN.

Therefore using only markers with statistical evidence of being associated with QTL increased the accuracy of GS for all traits except GYN regardless of the number of lines in the TP. Others have shown that selecting a greatly reduced subset of markers based on effects (Abdollahi-Arpanahi *et al.* 2014; Moser *et al.* 2010; Vazquez *et al.* 2010; Weigel *et al.* 2009) or consistency (Schulz-Streeck *et al.* 2013) produced GS accuracies that were similar to those obtained using all markers. Our results are unique as markers were selected based on significance and the subsets often produced increased accuracy relative to using all markers. The fact that marker subset 2 provides the highest prediction accuracies for most traits could be attributed to this marker set including enough markers to represent the genome while only including those with evidence of being linked to QTL controlling the trait. It should be noted that similar GS accuracies were obtained for all traits with marker subset 5 which were likely too small to cover the wheat genome, yet must be covering the most relevant regions of the genome. It is easy to understand why a model using only the significant markers is more predictive than a model using all markers as the effects associated with the selected markers are larger and more likely reflect the effects of real QTL than markers that are not significant. Including markers that are not significantly associated with a QTL lowered the accuracy perhaps because the effects associated with these regions are poorly estimated and thus these markers primarily add error or noise to the data set. In addition RRBLUP distributes variation equally over all markers and equally shrinks all marker effects toward zero (Meuwissen *et al.* 2001). By removing markers not statistically associated with a trait, perhaps the model can more accurately estimate marker effects and there is less shrinkage of true marker effects.

### GS accuracy between environments

Using data from one set of environments to predict the performance in independent environments is the crux of plant breeding. The GS prediction accuracy between environments for grain yield was very low (Table 1) when using all markers and lines. However, the accuracy of between-environment predictions for grain yield was increased considerably when using the subset of lines with low GEI and subsets of markers associated with grain yield in either environment. The highest prediction accuracy for grain yield between environments (*r* = 0.37) was found using the subset of 400 lines with low GEI and marker subset 6 (362 markers significant at *P* < 0.01 and with marker effects whose absolute value was >30 kg hectare^-1^, Table 1) and was greater than the phenotypic correlation of GYW and GYN (*r* = 0.20). The majority (99%) of the markers in subset 6 are not in linkage disequilibrium (LD) with one another suggesting these markers are likely mostly tagging independent QTL.

As with grain yield, the between-environment prediction accuracies for FHB, SE, and FY were lower than when using data from both years to build the model and it varied depending on which year’s data were used to build the model. For these traits using a subset of lines with low GEI had little impact on improving accuracy (Table 1). Using subsets of markers did improve the between-environment accuracy especially when using 2010 data to build the model to predict 2011 FY or SE phenotypes and using 2011 data to predict 2010 FHB phenotypes (Table 1). Thus, as for grain yield, the ability of the data from one environment to predict the other, and the impact of using subsets of TP data, varied by which environment’s data were used to build the prediction model. For every prediction scenario the greatest GS accuracy was attained by using either a subset of lines and/or a subset of markers (Table 1). The greatest GS accuracy between environments for FHB (*r* = 0.42) was greater than the correlation of FHB phenotypes between environments (*r* = 0.32). However, for SE the highest accuracy between environments was lower than the correlation of phenotypes between environments (*r* = 0.81) and for FY was equal to the correlation between environments (*r* = 0.72).

For grain yield, removing lines with high GEI likely allowed the model to more accurately estimate genetic effects and GEBVs. A line’s mean phenotype within an environment is due in part to its genetic effect and in part to the GEI of that line with the environment: the calculated GEI is due in part to true GEI but also in part to experimental error that may be confounded with that line and that environment. It is interesting that the accuracy of GYA and GYW decreased (relative to using all TP data) when using a subset of lines, but increased when using subsets of markers. In contrast, the GS accuracy for GYN and the between-environment GS accuracies only increased when using both the subset of lines and subsets of markers. This could be due to the error (noise) present in the phenotypic data for GYN, likely caused by the water damage to these plots. Most of the 70 lines with high GEI variance that were removed had negative GEI with the GYN environment and positive GEI with Wooster. This likely led to underestimating the true yield potential of these lines in the GYN environments while data from the Wooster environments were more indicative of their true genetic value. Including these high GEI lines in any analysis adds noise to the GYN data set and that reduces our ability to predict GYN, or the ability of GYN data to predict grain yield elsewhere. Thus GS accuracy is low for GYN and between-environment predictions when using data from all TP lines even with the reduced marker subsets. This is not a problem when using GYW or GYA data (GYW is a component of GYA) as the GYW phenotype data likely more accurately reflected true genetic values than did the GYN phenotypic data. Prediction accuracy for GYA and GYW increased using just subsets of markers as the markers were selected for significance for GYW or GYN, which means they were discerning genetic effects from noise.

The GS accuracy and RE_y_ observed for these traits in the TP indicate that GS could be very useful for improving these traits. This conclusion is further supported by the ability of the TP data and GS model to predict the performance of the parents of the TP. For all traits, the accuracy of GS can be greatly increased by using subsets of markers that are significant for the trait. The increase can be even greater when also using a subset of lines with low GEI for some traits. Increases of GS accuracy within the TP using cross-validation of up to 131% were attained by using data subsets. Even using marker subsets containing <7% of the total markers produced greater accuracies than using all markers. The increase in accuracy from using subsets of TP lines and/or markers is similar to what other studies have shown one would expect by increasing the TP size and number of markers (Bernardo and Yu 2007; Heffner *et al.* 2011a,b; Lorenz *et al.* 2012; Poland *et al.* 2012). Perhaps even more important is the finding that predicting the performance of lines in one set of environments by using only TP data from a second set of independent environments was significantly improved by using subsets of lines and markers.

It is now relatively easy and economical to generate lots of marker data using genotyping-by-sequence (GBS). However, our results suggest that a small subset of selected markers can provide more accurate GS predictions than a large number of markers. It is important to note than one must have a large number of markers and TP lines to begin with to have accurate estimates of genetic effects and genome coverage. From the large set of markers you can systematically select lines and markers to form a small and more informative subset that produces the greatest accuracy for use when implementing GS. These small marker subsets may allow the use of alternative genotyping platforms that are less expensive and more repeatable than GBS in future cycles of GS initiated from the TP. In plant breeding it is difficult to add more lines to a TP to increase the accuracy of GS as this greatly increases phenotyping resources. The TP size must allow for efficient phenotyping over multiple environments in order to accurately estimate GEI and marker effects. This study removed 5 to 17% of the TP lines based on GEI: the remaining lines must form a TP of sufficient size to provide robust estimates of genetic effects. These results suggest that starting with a large number of lines and markers in the TP allows one to select for the most informative ones to use in generating GS models with improved accuracy that can then be used when implementing GS.

## Supplementary Material

Supplemental Material
