# Supplementary material for: Optimizing Training Population Data and Validation of Genomic Selection for Economic Traits in Soft Winter Wheat
Source: G3 (Bethesda). 2016 Jul 20;6(9):2919–28. doi: 10.1534/g3.116.032532 (PMC5015948; doi:10.1534/g3.116.032532)
Supplement: Supplemental Material [file supp_6_9_2919__index.html]

Optimizing Training Population Data and Validation of Genomic Selection for Economic Traits in Soft Winter Wheat — Supplemental Material 

# Optimizing Training Population Data and Validation of Genomic Selection for Economic Traits in Soft Winter Wheat

## Supplemental Material for Hoffstetter *et al.*, 2016

**Files in this Data Supplement:**

- Table S1 - Summary of the entry, name, and pedigree of the 470 lines in the training population (TP). (.pdf, 112 KB)
- Table S2 - Summary of the 23 lines in the parental line population (PP). (.pdf, 14 KB)
- Table S3 - Summary of the 93 lines in the validation population (VP). (.pdf, 25 KB)
- Table S4 - Accuracy of genomic selection when using subsets of non-significant markers (M&, e.g., p > 0.05) as compared to the accuracy using all markers (M1), or just the significant markers (M2, p < 0.05). (.pdf, 21 KB)
