# Supplementary material for: Optimizing Training Population Data and Validation of Genomic Selection for Economic Traits in Soft Winter Wheat
Source: G3 (Bethesda). 2016 Jul 20;6(9):2919–28. doi: 10.1534/g3.116.032532 (PMC5015948; doi:10.1534/g3.116.032532)
Supplement: Supplemental Material [file supp_g3.116.032532_TableS1.pdf]

Table S1. Summary of the entry, name, and pedigree of the 470 lines in the training population (TP)

Entry	Name	Pedigree
1	OH08-198-22	VA98W-706 / IL96-6472
2	OH08-199-1	VA98W-706 / IL96-6472
3	OH08-199-73	VA98W-706 / IL96-6472
4	OH08-199-77	VA98W-706 / IL96-6472
5	OH08-200-11	VA98W-706 / IL96-6472
6	OH08-200-17	VA98W-706 / IL96-6472
7	OH08-200-25	VA98W-706 / IL96-6472
8	OH08-200-55	VA98W-706 / IL96-6472
9	OH08-200-7	VA98W-706 / IL96-6472
10	OH07-106-72	OH751 / BRAVO
11	OH07-107-27	OH751 / BRAVO
12	OH07-124-30	HONEY / BRAVO
13	OH07-124-37	HONEY / BRAVO
14	OH07-125-36	HONEY / BRAVO
15	OH07-125-55	HONEY / BRAVO
16	OH07-131-6	HONEY / OH708
17	OH07-147-22	HONEY / ROANE
18	OH07-148-12	HONEY / ROANE
19	OH07-161-29	OH708 / HOPEWELL
20	OH07-162-24	OH708 / HOPEWELL
21	OH07-174-62	OH708 / P.92145E8-7-7-1-9
22	OH07-189-72	ROANE / P.92145E8-7-7-1-9
23	OH07-191-41	ROANE / P.92145E8-7-7-1-9
24	OH07-238-15	P.92145E8-7-7-1-9 / TRIBUTE
25	OH07-238-53	P.92145E8-7-7-1-9 / TRIBUTE
26	OH07-259-12	HONEY / HOPEWELL
27	OH07-259-2	HONEY / HOPEWELL
28	OH07-259-28	HONEY / HOPEWELL
29	OH07-259-8	HONEY / HOPEWELL
30	OH07-262-25	HONEY / BRAVO
31	OH07-264-30	OH708 / P.92145E8-7-7-1-9
32	OH07-264-58	OH751 / BRAVO
33	OH07-264-70	OH751 / BRAVO
34	OH07-264-74	OH751 / BRAVO
35	OH07-74-15	HONEY / FREEDOM
36	OH07-74-24	HONEY / FREEDOM
37	OH07-74-44	HONEY / FREEDOM
38	OH07-75-1	HONEY / FREEDOM

39	OH07-75-10	HONEY / FREEDOM
40	OH07-75-45	HONEY / FREEDOM
41	OH07-78-11	ROANE / FREEDOM
42	OH07-78-27	ROANE / FREEDOM
43	OH07-80-59	ROANE / FREEDOM
44	OH07-85-25	PATTON / HOPEWELL
45	OH07-85-50	PATTON / HOPEWELL
46	OH07-85-59	PATTON / HOPEWELL
47	OH07-85-7	PATTON / HOPEWELL
48	OH07-86-37	PATTON / HOPEWELL
49	OH07-86-63	PATTON / HOPEWELL
50	OH07-86-73	PATTON / HOPEWELL
51	OH07-87-25	PATTON / HOPEWELL
52	OH07-124-66	HONEY / BRAVO
53	OH07-132-78	HONEY / OH708
54	OH07-131-73	HONEY / OH708
55	OH07-130-78	HONEY / OH708
56	OH07-131-4	HONEY / OH708
57	OH07-130-17	HONEY / OH708
58	OH07-148-21	HONEY / ROANE
59	OH07-148-42	HONEY / ROANE
60	OH07-148-1	HONEY / ROANE
61	OH07-147-26	HONEY / ROANE
62	OH07-147-30	HONEY / ROANE
63	OH07-160-52	OH708 / HOPEWELL
64	OH07-160-61	OH708 / HOPEWELL
65	OH07-160-72	OH708 / HOPEWELL
66	OH07-162-35	OH708 / HOPEWELL
67	OH07-161-62	OH708 / HOPEWELL
68	OH07-162-65	OH708 / HOPEWELL
69	OH07-159-62	OH708 / HOPEWELL
70	OH07-162-17	OH708 / HOPEWELL
71	OH07-162-12	OH708 / HOPEWELL
72	OH07-161-56	OH708 / HOPEWELL
73	OH07-174-77	OH708 / P.92145E8-7-7-1-9
74	OH07-175-34	OH708 / P.92145E8-7-7-1-9
75	OH07-264-49	OH708 / P.92145E8-7-7-1-9
76	OH07-264-35	OH708 / P.92145E8-7-7-1-9
77	OH07-176-46	OH708 / P.92145E8-7-7-1-9
78	OH07-173-28	OH708 / P.92145E8-7-7-1-9
79	OH07-175-8	OH708 / P.92145E8-7-7-1-9
80	OH07-173-64	OH708 / P.92145E8-7-7-1-9
81	OH07-173-56	OH708 / P.92145E8-7-7-1-9
82	OH07-174-11	OH708 / P.92145E8-7-7-1-9
83	OH07-175-73	OH708 / P.92145E8-7-7-1-9

84	OH07-176-6	OH708 / P.92145E8-7-7-1-9
85	OH07-173-50	OH708 / P.92145E8-7-7-1-9
86	OH07-176-56	OH708 / P.92145E8-7-7-1-9
87	OH07-175-11	OH708 / P.92145E8-7-7-1-9
88	OH07-174-50	OH708 / P.92145E8-7-7-1-9
89	OH07-240-55	P.92145E8-7-7-1-9 / TRIBUTE
90	OH07-237-12	P.92145E8-7-7-1-9 / TRIBUTE
91	OH07-238-14	P.92145E8-7-7-1-9 / TRIBUTE
92	OH07-240-66	P.92145E8-7-7-1-9 / TRIBUTE
93	OH07-237-45	P.92145E8-7-7-1-9 / TRIBUTE
94	OH07-80-74	ROANE / FREEDOM
95	OH07-88-63	ROANE / HOPEWELL
96	OH07-88-53	ROANE / HOPEWELL
97	OH07-89-22	ROANE / HOPEWELL
98	OH07-89-57	ROANE / HOPEWELL
99	OH07-258-27	ROANE / P.92145E8-7-7-1-9
100	OH07-190-10	ROANE / P.92145E8-7-7-1-9
101	OH07-191-66	ROANE / P.92145E8-7-7-1-9
102	OH07-191-70	ROANE / P.92145E8-7-7-1-9
103	OH07-190-56	ROANE / P.92145E8-7-7-1-9
104	OH07-189-56	ROANE / P.92145E8-7-7-1-9
105	OH07-190-42	ROANE / P.92145E8-7-7-1-9
106	OH07-191-45	ROANE / P.92145E8-7-7-1-9
107	OH07-189-28	ROANE / P.92145E8-7-7-1-9
108	OH07-188-68	ROANE / P.92145E8-7-7-1-9
109	OH07-95-19	ROANE / PATTON
110	OH07-94-6	ROANE / PATTON
111	OH07-95-58	ROANE / PATTON
112	OH07-94-70	ROANE / PATTON
113	OH07-93-15	ROANE / PATTON
114	OH07-95-7	ROANE / PATTON
115	OH07-93-62	ROANE / PATTON
116	OH07-95-33	ROANE / PATTON
117	OH07-174-17	OH708 / P.92145E8-7-7-1-9
118	OH07-190-12	ROANE / P.92145E8-7-7-1-9
119	OH07-191-62	ROANE / P.92145E8-7-7-1-9
120	OH07-258-42	ROANE / P.92145E8-7-7-1-9
121	OH08-100-38	TRUMAN / IL97-3632
122	OH08-100-74	TRUMAN / IL97-3632
123	OH08-101-34	TRUMAN / IL96-6472
124	OH08-101-38	TRUMAN / IL96-6472
125	OH08-101-57	TRUMAN / IL96-6472
126	OH08-101-72	TRUMAN / IL96-6472
127	OH08-102-18	TRUMAN / IL96-6472
128	OH08-102-45	TRUMAN / IL96-6472

129	OH08-103-12	TRUMAN / IL96-6472
130	OH08-103-70	TRUMAN / IL96-6472
131	OH08-104-32	TRUMAN / IL96-6472
132	OH08-104-41	TRUMAN / IL96-6472
133	OH08-106-15	TRUMAN / OH751
134	OH08-106-50	TRUMAN / OH751
135	OH08-106-66	TRUMAN / OH751
136	OH08-106-67	TRUMAN / OH751
137	OH08-107-16	TRUMAN / OH751
138	OH08-107-38	TRUMAN / OH751
139	OH08-107-4	TRUMAN / OH751
140	OH08-130-4	HOPEWELL / BRAVO
141	OH08-130-54	HOPEWELL / BRAVO
142	OH08-130-76	HOPEWELL / BRAVO
143	OH08-131-11	HOPEWELL / BRAVO
144	OH08-131-15	HOPEWELL / BRAVO
145	OH08-131-56	HOPEWELL / BRAVO
146	OH08-134-12	HONEY / FREEDOM
147	OH08-107-46	TRUMAN / OH751
148	OH08-107-50	TRUMAN / OH751
149	OH08-107-8	TRUMAN / OH751
150	OH08-134-39	HONEY / FREEDOM
151	OH08-134-52	HONEY / FREEDOM
152	OH08-135-13	HONEY / FREEDOM
153	OH08-135-64	HONEY / FREEDOM
154	OH08-136-10	HONEY / FREEDOM
155	OH08-136-16	HONEY / FREEDOM
156	OH08-136-18	HONEY / FREEDOM
157	OH08-136-43	HONEY / FREEDOM
158	OH08-156-69	ROANE / PATTON
159	OH08-156-7	ROANE / PATTON
160	OH08-141-33	HONEY / ROANE
161	OH08-141-6	HONEY / ROANE
162	OH08-142-25	PATTERSON / HOPEWELL
163	OH08-142-43	PATTERSON / HOPEWELL
164	OH08-142-63	PATTERSON / HOPEWELL
165	OH08-143-26	PATTERSON / HOPEWELL
166	OH08-143-62	PATTERSON / HOPEWELL
167	OH08-144-23	PATTERSON / HOPEWELL
168	OH08-144-7	PATTERSON / HOPEWELL
169	OH08-146-1	ROANE / HOPEWELL
170	OH08-146-27	ROANE / HOPEWELL
171	OH08-146-43	ROANE / HOPEWELL
172	OH08-146-51	ROANE / HOPEWELL
173	OH08-161-4	OH751 / OH738

174	OH08-161-55	OH751 / OH738
175	OH08-161-78	OH751 / OH738
176	OH08-162-45	OH751 / OH738
177	OH08-162-66	OH751 / OH738
178	OH08-162-75	OH751 / OH738
179	OH08-163-16	OH751 / OH738
180	OH08-163-54	OH751 / OH738
181	OH08-163-62	OH751 / OH738
182	OH08-168-24	DOUGLAS / IL97-3632
183	OH08-168-38	DOUGLAS / IL97-3632
184	OH08-168-4	DOUGLAS / IL97-3632
185	OH08-146-59	ROANE / HOPEWELL
186	OH08-147-13	ROANE / HOPEWELL
187	OH08-147-19	ROANE / HOPEWELL
188	OH08-147-3	ROANE / HOPEWELL
189	OH08-147-65	ROANE / HOPEWELL
190	OH08-168-75	DOUGLAS / IL97-3632
191	OH08-169-13	DOUGLAS / IL97-3632
192	OH08-169-48	DOUGLAS / IL97-3632
193	OH08-170-66	DOUGLAS / IL97-3632
194	OH08-174-31	DOUGLAS / OH751
195	OH08-153-43	PATTON / PATTERSON
196	OH08-153-49	PATTON / PATTERSON
197	OH08-154-21	PATTON / PATTERSON
198	OH08-154-59	PATTON / PATTERSON
199	OH08-154-9	PATTON / PATTERSON
200	OH08-155-1	ROANE / PATTON
201	OH08-155-16	ROANE / PATTON
202	OH08-155-74	ROANE / PATTON
203	OH08-155-9	ROANE / PATTON
204	OH08-156-13	ROANE / PATTON
205	OH08-156-30	ROANE / PATTON
206	OH08-156-55	ROANE / PATTON
207	OH08-174-55	DOUGLAS / OH751
208	OH08-175-49	DOUGLAS / OH751
209	OH08-175-50	DOUGLAS / OH751
210	OH08-175-70	DOUGLAS / OH751
211	OH08-176-31	DOUGLAS / OH751
212	OH08-177-27	DOUGLAS / P.92226E2-5-3
213	OH08-177-9	DOUGLAS / P.92226E2-5-3
214	OH08-178-52	DOUGLAS / P.92226E2-5-3
215	OH08-178-63	DOUGLAS / P.92226E2-5-3
216	OH08-178-75	DOUGLAS / P.92226E2-5-3
217	OH08-179-14	DOUGLAS / P.92226E2-5-3
218	OH08-179-28	DOUGLAS / P.92226E2-5-3

219	OH08-179-69	DOUGLAS / P.92226E2-5-3
220	OH08-194-30	VA98W-706 / OH751
221	OH08-194-38	VA98W-706 / OH751
222	OH08-194-71	VA98W-706 / OH751
223	OH08-194-8	VA98W-706 / OH751
224	OH08-195-28	VA98W-706 / OH751
225	OH08-196-25	VA98W-706 / OH751
226	OH08-196-66	VA98W-706 / OH751
227	OH08-196-69	VA98W-706 / OH751
228	OH08-197-23	VA98W-706 / OH751
229	OH08-197-35	VA98W-706 / OH751
230	OH08-197-52	VA98W-706 / OH751
231	OH08-198-52	VA98W-706 / IL96-6472
232	OH08-198-66	VA98W-706 / IL96-6472
233	OH08-199-18	VA98W-706 / IL96-6472
234	OH08-201-18	P.92226E2-5-3 / HONEY
235	OH08-182-4	DOUGLAS / OH708
236	OH08-182-43	DOUGLAS / OH708
237	OH08-182-46	DOUGLAS / OH708
238	OH08-183-13	DOUGLAS / OH708
239	OH08-184-15	VA98W-706 / P.92226E2-5-3
240	OH08-184-17	VA98W-706 / P.92226E2-5-3
241	OH08-184-41	VA98W-706 / P.92226E2-5-3
242	OH08-184-47	VA98W-706 / P.92226E2-5-3
243	OH08-184-50	VA98W-706 / P.92226E2-5-3
244	OH08-184-78	VA98W-706 / P.92226E2-5-3
245	OH08-185-18	VA98W-706 / P.92226E2-5-3
246	OH08-185-43	VA98W-706 / P.92226E2-5-3
247	OH08-185-56	VA98W-706 / P.92226E2-5-3
248	OH08-185-66	VA98W-706 / P.92226E2-5-3
249	OH08-185-7	VA98W-706 / P.92226E2-5-3
250	OH08-185-70	VA98W-706 / P.92226E2-5-3
251	OH08-185-72	VA98W-706 / P.92226E2-5-3
252	OH08-186-32	VA98W-706 / P.92226E2-5-3
253	OH08-201-39	P.92226E2-5-3 / HONEY
254	OH08-202-14	P.92226E2-5-3 / HONEY
255	OH08-202-49	P.92226E2-5-3 / HONEY
256	OH08-202-58	P.92226E2-5-3 / HONEY
257	OH08-202-73	P.92226E2-5-3 / HONEY
258	OH08-203-14	P.92226E2-5-3 / HONEY
259	OH08-203-31	P.92226E2-5-3 / HONEY
260	OH08-203-5	P.92226E2-5-3 / HONEY
261	OH08-203-55	P.92226E2-5-3 / HONEY
262	OH08-204-22	P.92226E2-5-3 / HONEY
263	OH08-204-47	P.92226E2-5-3 / HONEY

264	OH08-204-50	P.92226E2-5-3 / HONEY
265	OH08-204-70	P.92226E2-5-3 / HONEY
266	OH08-204-8	P.92226E2-5-3 / HONEY
267	OH08-205-13	P.92226E2-5-3 / OH751
268	OH08-186-43	VA98W-706 / P.92226E2-5-3
269	OH08-187-15	VA98W-706 / HONEY
270	OH08-187-27	VA98W-706 / HONEY
271	OH08-187-59	VA98W-706 / HONEY
272	OH08-188-13	VA98W-706 / HONEY
273	OH08-190-1	VA98W-706 / IL97-3632
274	OH08-190-10	VA98W-706 / IL97-3632
275	OH08-190-22	VA98W-706 / IL97-3632
276	OH08-190-25	VA98W-706 / IL97-3632
277	OH08-190-48	VA98W-706 / IL97-3632
278	OH08-191-2	VA98W-706 / IL97-3632
279	OH08-191-31	VA98W-706 / IL97-3632
280	OH08-191-32	VA98W-706 / IL97-3632
281	OH08-191-42	VA98W-706 / IL97-3632
282	OH08-191-7	VA98W-706 / IL97-3632
283	OH08-191-76	VA98W-706 / IL97-3632
284	OH08-191-78	VA98W-706 / IL97-3632
285	OH08-205-19	P.92226E2-5-3 / OH751
286	OH08-205-21	P.92226E2-5-3 / OH751
287	OH08-205-36	P.92226E2-5-3 / OH751
288	OH08-205-75	P.92226E2-5-3 / OH751
289	OH08-206-19	P.92226E2-5-3 / OH751
290	OH08-206-67	P.92226E2-5-3 / OH751
291	OH08-206-69	P.92226E2-5-3 / OH751
292	OH08-206-74	P.92226E2-5-3 / OH751
293	OH08-206-8	P.92226E2-5-3 / OH751
294	OH08-207-12	P.92226E2-5-3 / OH751
295	OH08-207-3	P.92226E2-5-3 / OH751
296	OH08-207-31	P.92226E2-5-3 / OH751
297	OH08-207-33	P.92226E2-5-3 / OH751
298	OH08-207-41	P.92226E2-5-3 / OH751
299	OH08-192-23	VA98W-706 / IL97-3632
300	OH08-192-42	VA98W-706 / IL97-3632
301	OH08-192-50	VA98W-706 / IL97-3632
302	OH08-192-67	VA98W-706 / IL97-3632
303	OH08-193-13	VA98W-706 / IL97-3632
304	OH08-193-42	VA98W-706 / IL97-3632
305	OH08-193-61	VA98W-706 / IL97-3632
306	OH08-194-17	VA98W-706 / OH751
307	OH08-194-2	VA98W-706 / OH751
308	OH08-223-53	IL98-4364 / OH740

309	OH08-224-15	IL98-4364 / OH740
310	OH08-224-75	IL98-4364 / OH740
311	OH08-225-32	IL98-4364 / OH751
312	OH08-225-71	IL98-4364 / OH751
313	OH08-226-22	IL98-4364 / OH751
314	OH08-226-26	IL98-4364 / OH751
315	OH08-226-59	IL98-4364 / OH751
316	OH08-207-56	P.92226E2-5-3 / OH751
317	OH08-207-58	P.92226E2-5-3 / OH751
318	OH08-207-73	P.92226E2-5-3 / OH751
319	OH08-207-77	P.92226E2-5-3 / OH751
320	OH08-208-14	P.92226E2-5-3 / OH751
321	OH08-208-44	P.92226E2-5-3 / OH751
322	OH08-208-70	P.92226E2-5-3 / OH751
323	OH08-209-15	P.92226E2-5-3 / OH751
324	OH08-209-42	P.92226E2-5-3 / OH751
325	OH08-209-6	P.92226E2-5-3 / OH751
326	OH08-209-71	P.92226E2-5-3 / OH751
327	OH08-227-24	OH738 / HONEY
328	OH08-227-49	OH738 / HONEY
329	OH08-228-30	OH738 / HONEY
330	OH08-229-49	OH738 / HONEY
331	OH08-229-63	OH738 / HONEY
332	OH08-231-21	OH738 / CECIL
333	OH08-232-44	OH738 / CECIL
334	OH08-232-70	OH738 / CECIL
335	OH08-233-19	OH738 / CECIL
336	OH08-234-2	OH738 / OH740
337	OH08-234-32	OH738 / OH740
338	OH08-234-37	OH738 / OH740
339	OH08-234-4	OH738 / OH740
340	OH08-234-54	OH738 / OH740
341	OH08-235-11	OH738 / OH740
342	OH08-209-75	P.92226E2-5-3 / OH751
343	OH08-210-14	P.92226E2-5-3 / MCCORMICK
344	OH08-210-27	P.92226E2-5-3 / MCCORMICK
345	OH08-210-51	P.92226E2-5-3 / MCCORMICK
346	OH08-210-55	P.92226E2-5-3 / MCCORMICK
347	OH08-210-75	P.92226E2-5-3 / MCCORMICK
348	OH08-211-51	P.92226E2-5-3 / MCCORMICK
349	OH08-211-70	P.92226E2-5-3 / MCCORMICK
350	OH08-211-74	P.92226E2-5-3 / MCCORMICK
351	OH08-212-15	P.92226E2-5-3 / MCCORMICK
352	OH08-212-31	P.92226E2-5-3 / MCCORMICK
353	OH08-212-46	P.92226E2-5-3 / MCCORMICK

354	OH08-216-10	IL98-4364 / HONEY
355	OH08-235-33	OH738 / OH740
356	OH08-237-19	OH751 / OH738
357	OH08-238-4	P.961341A3-2-2 / HONEY
358	OH08-216-49	IL98-4364 / HONEY
359	OH08-216-57	IL98-4364 / HONEY
360	OH08-217-34	IL98-4364 / HONEY
361	OH08-217-37	IL98-4364 / HONEY
362	OH08-217-74	IL98-4364 / HONEY
363	OH08-217-9	IL98-4364 / HONEY
364	OH08-218-37	IL98-4364 / HONEY
365	OH08-218-6	IL98-4364 / HONEY
366	OH08-219-66	IL98-4364 / HONEY
367	OH08-220-43	IL98-4364 / CECIL
368	OH08-220-68	IL98-4364 / CECIL
369	OH08-221-27	IL98-4364 / CECIL
370	OH08-221-50	IL98-4364 / CECIL
371	OH08-221-63	IL98-4364 / CECIL
372	OH08-222-16	IL98-4364 / CECIL
373	OH08-223-25	IL98-4364 / OH740
374	OH08-239-31	P.961341A3-2-2 / HONEY
375	OH08-239-35	P.961341A3-2-2 / HONEY
376	OH08-239-63	P.961341A3-2-2 / HONEY
377	OH08-239-67	P.961341A3-2-2 / HONEY
378	OH08-240-31	P.961341A3-2-2 / HONEY
379	OH08-240-46	P.961341A3-2-2 / HONEY
380	OH08-241-22	P.961341A3-2-2 / HONEY
381	OH08-241-63	P.961341A3-2-2 / HONEY
382	OH08-241-67	P.961341A3-2-2 / HONEY
383	OH08-242-15	P.961341A3-2-2 / CECIL
384	OH08-242-30	P.961341A3-2-2 / CECIL
385	OH08-253-35	MCCORMICK / OH751
386	OH08-253-41	MCCORMICK / OH751
387	OH08-254-22	TRIBUTE / P.92226E2-5-3
388	OH08-254-24	TRIBUTE / P.92226E2-5-3
389	OH08-254-28	TRIBUTE / P.92226E2-5-3
390	OH08-254-6	TRIBUTE / P.92226E2-5-3
391	OH08-254-66	TRIBUTE / P.92226E2-5-3
392	OH08-255-29	TRIBUTE / P.92226E2-5-3
393	OH08-256-31	TRIBUTE / P.92226E2-5-3
394	OH08-242-31	P.961341A3-2-2 / CECIL
395	OH08-242-38	P.961341A3-2-2 / CECIL
396	OH08-242-51	P.961341A3-2-2 / CECIL
397	OH08-242-71	P.961341A3-2-2 / CECIL
398	OH08-242-78	P.961341A3-2-2 / CECIL

399	OH08-243-15	P.961341A3-2-2 / CECIL
400	OH08-243-23	P.961341A3-2-2 / CECIL
401	OH08-243-31	P.961341A3-2-2 / CECIL
402	OH08-243-46	P.961341A3-2-2 / CECIL
403	OH08-244-10	P.961341A3-2-2 / CECIL
404	OH08-244-2	P.961341A3-2-2 / CECIL
405	OH08-244-28	P.961341A3-2-2 / CECIL
406	OH08-244-52	P.961341A3-2-2 / CECIL
407	OH08-244-57	P.961341A3-2-2 / CECIL
408	OH08-245-24	P.961341A3-2-2 / OH740
409	OH08-256-35	TRIBUTE / P.92226E2-5-3
410	OH08-256-47	TRIBUTE / P.92226E2-5-3
411	OH08-256-66	TRIBUTE / P.92226E2-5-3
412	OH08-257-27	TRIBUTE / P.92226E2-5-3
413	OH08-262-27	ROANE / HOPEWELL
414	OH08-262-36	ROANE / HOPEWELL
415	OH08-262-42	ROANE / HOPEWELL
416	OH08-262-55	ROANE / HOPEWELL
417	OH08-245-47	P.961341A3-2-2 / OH740
418	OH08-245-5	P.961341A3-2-2 / OH740
419	OH08-245-55	P.961341A3-2-2 / OH740
420	OH08-246-10	P.961341A3-2-2 / OH740
421	OH08-246-15	P.961341A3-2-2 / OH740
422	OH08-247-15	P.961341A3-2-2 / OH740
423	OH08-247-3	P.961341A3-2-2 / OH740
424	OH08-247-39	P.961341A3-2-2 / OH740
425	OH08-247-48	P.961341A3-2-2 / OH740
426	OH08-247-77	P.961341A3-2-2 / OH740
427	OH08-248-23	P.961341A3-2-2 / OH740
428	OH08-263-3	DOUGLAS / IL97-3632
429	OH08-263-34	DOUGLAS / OH751
430	OH08-264-24	MCCORMICK / OH751
431	OH08-264-29	MCCORMICK / OH751
432	OH08-264-67	MCCORMICK / OH751
433	OH08-265-11	DOUGLAS / P.92226E2-5-3
434	OH08-265-13	DOUGLAS / P.92226E2-5-3
435	OH08-265-37	DOUGLAS / P.92226E2-5-3
436	OH08-265-45	DOUGLAS / P.92226E2-5-3
437	OH08-248-54	P.961341A3-2-2 / OH740
438	OH08-248-62	P.961341A3-2-2 / OH740
439	OH08-248-70	P.961341A3-2-2 / OH740
440	OH08-248-77	P.961341A3-2-2 / OH740
441	OH08-248-8	P.961341A3-2-2 / OH740
442	OH08-268-39	P.961341A3-2-2 / CECIL
443	OH08-268-56	TRIBUTE / P.92226E2-5-3

444	OH08-268-74	TRIBUTE / P.92226E2-5-3
445	OH08-266-27	VA98W-706 / IL97-3632
446	OH08-266-9	VA98W-706 / IL97-3632
447	OH08-267-14	P.961341A3-2-2 / HONEY
448	OH08-267-16	P.961341A3-2-2 / HONEY
449	OH08-267-25	P.961341A3-2-2 / HONEY
450	OH08-267-3	P.961341A3-2-2 / HONEY
451	OH08-267-34	P.961341A3-2-2 / HONEY
452	OH08-268-11	P.961341A3-2-2 / CECIL
453	OH08-268-23	P.961341A3-2-2 / CECIL
454	OH08-268-30	P.961341A3-2-2 / CECIL
455	OH08-269-6	P.92226E2-5-3 / OH751
456	OH08-3-29	TRUMAN / OH751
457	OH08-3-35	TRUMAN / OH751
458	OH08-3-41	TRUMAN / OH751
459	OH08-97-24	TRUMAN / IL97-3632
460	OH08-97-47	TRUMAN / IL97-3632
461	OH08-97-9	TRUMAN / IL97-3632
462	OH08-98-13	TRUMAN / IL97-3632
463	OH08-98-29	TRUMAN / IL97-3632
464	OH08-98-6	TRUMAN / IL97-3632
465	OH08-98-75	TRUMAN / IL97-3632
466	OH08-99-23	TRUMAN / IL97-3632
467	OH08-99-25	TRUMAN / IL97-3632
468	OH08-99-31	TRUMAN / IL97-3632
469	OH08-99-41	TRUMAN / IL97-3632
470	OH08-99-50	TRUMAN / IL97-3632
