# Supplementary material for: Optimizing Training Population Data and Validation of Genomic Selection for Economic Traits in Soft Winter Wheat
Source: G3 (Bethesda). 2016 Jul 20;6(9):2919–28. doi: 10.1534/g3.116.032532 (PMC5015948; doi:10.1534/g3.116.032532)
Supplement: Supplemental Material [file supp_g3.116.032532_TableS2.pdf]

Table S2. Summary of the 23 lines in the parental line population (PP). * indicates a parent that was not included in the analysis due to a lack of seed.

Name	% of training population parentage	Pedigree
ERIE (OH751)	9.3	10584-0801/COKER9663
HOPEWELL	5.7	LOGAN/HART//3270A/ROUSSALKA/3/TN1685/IA22/6767/216-6-3
CECIL	3.3	BLUEBOY2/CLARK//HOWELL/OH416
OH708	3.2	IL85-3132-1/IRENA//OH449/VA85-54-290
OH740*	3.1	L89060/OH529
OH738	2.9	L890690/T814
FREEDOM	2.0	GR876/OH217
HONEY	9.8	DYNASTY/3/NASW84-345/COKER9835//OH419/OH389
PATTON	3.4	SW85*94/IN82104B1-3-2
DOUGLAS	3.1	Pio.2510/M92*3449
BRAVO	1.9	0
92226E2-5-3	10.3	86982/844030//86350/831800
961341A3-2-2	5.8	P9017//INW9824/3/P107/PATTERSON
92145E8-7-7-1-9	3.4	COKER84-27/COTIPORA//ROAZON/CALDWELL*2
PATTERSON	1.2	P.69184B8-21-1-1-2-4*2/CALDWELL
IL97-3632	4.5	Howell/IL90-9464//IL90-4813
IL98-4364	2.8	OH530/Patterson
IL96-6472	2.4	IL90-11637/L880437
TRUMAN	4.1	MO11769/MADISON
VA98W-706	7	VA91-54-350//FFR555W/Gore
ROANE	6.9	VA71-54-147/COKER68-15//IN65309C1-18-2-3-2
TRIBUTE	2.1	IN71761A4-31-5-48//VA71-54-147/MCNAIR1813)/AL870365(COKER747*2/AMIGO)
MCCORMICK	1.8	VA92-51-39(IN71761A4-31-5-48//VA71-54-147/MCNAIR1813)/AL870365(COKER747*2/AMIGO)
