# Supplementary material for: Optimizing Training Population Data and Validation of Genomic Selection for Economic Traits in Soft Winter Wheat
Source: G3 (Bethesda). 2016 Jul 20;6(9):2919–28. doi: 10.1534/g3.116.032532 (PMC5015948; doi:10.1534/g3.116.032532)
Supplement: Supplemental Material [file supp_g3.116.032532_TableS3.pdf]

Table S3. Summary of the 93 lines in the validation population (VP)

Name	Pedigree
OH10-306-43	981359C1-4-2-1-8/BRANSON
OH10-308-46	981359C1-4-2-1-8/M99-2408
OH10-123-3	9819RB-D1-30/OH02-13581
OH10-151-70	B980582/OH02-12686
OH10-153-41	B980582/OH753
OH10-155-35	B980582/OH776
OH10-156-2	B980582/P.99608C1-1-3
OH10-158-62	B980582/P.99608C1-1-3
OH10-158-69	B980582/P.99608C1-1-3
OH10-317-7	B980582/99608C1-1-3
OH10-185-30	BRANSON/B980582
OH10-194-16	BRANSON/IL00-8061
OH10-194-33	BRANSON/IL00-8061
OH10-194-46	BRANSON/IL00-8061
OH10-198-15	BRANSON/IL96-24851-1
OH10-200-49	BRANSON/IL96-24851-1
OH10-201-32	BRANSON/M99-2408
OH10-201-65	BRANSON/M99-2408
OH10-203-55	BRANSON/M99-2408
OH10-204-59	BRANSON/M99-2408
OH10-186-24	BRANSON/B980582
OH10-213-21	BRANSON/OH02-3409
OH10-222-78	BRANSON/TRUMAN
OH10-223-2	BRANSON/TRUMAN
OH10-308-23	BRANSON/M99-2408
OH10-308-27	BRANSON/M99-2408
OH10-303-54	BRANSON/TRUMAN
OH08-133-25	HONEY/COKER9663
OH08-132-51	HONEY/COKER9663
OH08-133-14	HONEY/COKER9663
OH08-133-41	HONEY/COKER9663
OH07-136-66	HONEY/OH719
OH10-167-30	IL00-8061/M99-2408
OH10-250-19	IL00-8061/MO980829
OH10-304-30	IL00-8061/M99-2408
OH10-304-42	IL00-8061/M99-2408
OH10-304-71	IL00-8061/M99-2408
OH10-304-27	IL00-8061/M99-2408
OH10-104-31	IL96-24851-1/IL00-8061
OH10-94-23	IL96-24851-1/M99-2408
OH10-95-17	IL96-24851-1/M99-2408
OH10-316-61	IL96-24851-1/M99-2408

OH10-173-55	IL97-6268/B980582
OH10-179-19	IL97-6268/OH776
OH10-179-35	IL97-6268/OH776
OH10-182-75	IL97-6268/P.99608C1-1-3
OH10-227-31	M99-2408/CECIL
OH10-231-26	M99-2408/IL97-6268
OH10-231-54	M99-2408/IL97-6268
OH10-231-6	M99-2408/IL97-6268
OH10-237-51	M99-2408/OH01-75
OH10-238-15	M99-2408/OH01-75
OH10-238-59	M99-2408/OH01-75
OH10-239-28	M99-2408/OH02-12686
OH10-240-53	M99-2408/OH02-12686
OH10-240-6	M99-2408/OH02-12686
OH10-241-49	M99-2408/OH02-12686
OH10-241-53	M99-2408/OH02-12686
OH10-242-45	M99-2408/OH02-12686
OH10-244-27	M99-2408/OH753
OH10-101-54	M99-2408/P.981359C1-4-2-1-8
OH10-103-72	M99-2408/P.981359C1-4-2-1-8
OH10-245-63	M99-2408/P.99608C1-1-3
OH10-246-14	M99-2408/P.99608C1-1-3
OH10-246-26	M99-2408/P.99608C1-1-3
OH10-247-15	M99-2408/P.99608C1-1-3
OH10-305-76	M99-2408/981359C1-4-2-1-8
OH10-305-39	M99-2408/P.981359C1-4-2-1-8
OH10-305-47	M99-2408/P.981359C1-4-2-1-8
OH10-305-55	M99-2408/P.981359C1-4-2-1-8
OH10-305-59	M99-2408/P.981359C1-4-2-1-8
OH10-315-5	M99-2408/OH02-12686
OH10-316-14	M99-2408/OH02-12686
OH10-316-20	M99-2408/OH02-12686
OH10-316-22	M99-2408/OH02-12686
OH10-292-7	OH02-12686/P.981359C1-4-2-1-8
OH10-300-21	OH02-12686/MO980829
OH10-306-22	OH708/99608C1-1-3
OH10-271-34	OH708/IL97-6268
OH10-204-78	BRANSON/M99-2408
OH07-261-37	OH728/VA97W-361WS
OH10-128-47	OH776/9819RB-D1-42
OH10-307-9	99608C1-1-3/BRANSON
OH10-219-65	P.99608C1-1-3/BRANSON
OH10-220-3	P.99608C1-1-3/BRANSON
OH10-221-64	P.99608C1-1-3/BRANSON
OH10-90-12	TRUMAN/IL00-8061

OH10-91-17	TRUMAN/IL00-8061
OH10-91-37	TRUMAN/IL00-8061
OH10-91-54	TRUMAN/IL00-8061
OH10-92-23	TRUMAN/IL00-8061
OH10-92-55	TRUMAN/IL00-8061
OH10-92-78	TRUMAN/IL00-8061
OH10-301-45	TRUMAN/IL00-8061
