# Supplementary material for: Optimizing Training Population Data and Validation of Genomic Selection for Economic Traits in Soft Winter Wheat
Source: G3 (Bethesda). 2016 Jul 20;6(9):2919–28. doi: 10.1534/g3.116.032532 (PMC5015948; doi:10.1534/g3.116.032532)
Supplement: Supplemental Material [file supp_g3.116.032532_TableS4.pdf]

Table S4. Accuracy of genomic selection when using subsets of non-significant markers (M&, e.g., p > 0.05) as compared to the accuracy using all markers (M1), or just the significant markers (M2, p < 0.05).

Trait	All Markers = M1	Markers with p <0.05 = M3	Markers with p > 0.05 = M7	M7 as % of M1	M7 as % of M2
No. Markers for GYA, GYW, GYN	33169	2902	30267		
GYA, n=470	0.46	0.77	0.33	71.7	42.9
GYA, n=400	0.45	0.82	0.31	68.9	37.8
GYW, n=470	0.57	0.79	0.48	84.2	60.8
GYW, n=400	0.54	0.60	0.44	81.5	73.3
GYN, n=470	0.43	0.36	0.43	100.0	119.4
GYN, n=400	0.33	0.77	0.23	69.7	29.9
GYW to predict GYN, n=470	-0.08	-0.10	-0.07		
GYW to predict GYN, n=400	0.16	0.31	0.07	43.8	22.6
GYN to predict GYW, n=470	-0.11	-0.16	-0.09		
GYN to predict GYW, n=400	0.19	0.28	0.11	57.9	39.3
No. Markers for FHB	33169	1556	31613		
FHB, n=470	0.35	0.64	0.28	80.0	43.8
FHB, n=440	0.37	0.81	0.26	70.3	32.1
2010 FHB to predict 2011 FHB, n=470	0.15	0.17	0.13	86.7	76.5
2010 FHB to predict 2011 FHB, n=440	0.15	0.17	0.14	93.3	82.4
2011 FHB to predict 2010 FHB, n=470	0.15	0.32	0.13	86.7	40.6
2011 FHB to predict 2010 FHB, n=440	0.17	0.38	0.14	82.4	36.8
No. Markers for SE	33169	1672	31537		
SE, n=470	0.51	0.87	0.55	107.8	63.2
SE, n=447	0.51	0.89	0.55	107.8	61.8
2010 SE to predict 2011 SE, n=470	0.33	0.57	0.55	166.7	96.5
2010 SE to predict 2011 SE, n=447	0.33	0.58	0.58	175.8	100.0
2011 SE to predict 2010 SE, n=470	0.24	0.32	0.46	191.7	143.8
2011 SE to predict 2010 SE, n=447	0.24	0.32	0.47	195.8	146.9
No. Markers for FY	33169	1632	31497		
FY, n=470	0.62	0.91	0.4	64.5	44.0
FY, n=447	0.64	0.91	0.39	60.9	42.9
2010 FY to predict 2011 FY, n=470	0.57	0.70	0.29	50.9	41.4
2010 FY to predict 2011 FY, n=447	0.61	0.74	0.29	47.5	39.2
2011 FY to predict 2010 FY, n=470	0.48	0.49	0.21	43.8	42.9
2011 FY to predict 2010 FY, n=447	0.49	0.50	0.21	42.9	42.0
